# Puerperal Fever

**Published:** 1833

**Authors:** 


### II. ANALYTICAL.

Art. I. *Puerperal Fever.*—In the last number of our eastern contemporary, the American Journal, there is a long practical paper, by our friend Dr. Hugh L. Hodge, on Puerperal Fever. The subject is one of such great importance, and we have within the last few days been so painfully interested in a case of this disease, that we are induced, notwithstanding the Doctor's paper contains nothing particularly new, either in pathology or therapeutics, to present our readers with a liberal extract.

"The great fatality of puerperal peritonitis, whether appearing sporadically or epidemically, in private or public practice, is well known. When endemic in hospitals few patients recover—not unfrequently almost every individual perishes. This mortality no doubt partially depends on the predisposing causes, of which, as already acknowledged, we are very ignorant, but which are evidently more influential in hospitals, and other similar situations, and modify, in a most important manner, the symptoms and progress of the disease, as I shall attempt to demonstrate, rendering similar modification in the treatment essentially necessary. The very nature of puerperal fever, being an acute imflammation of a most irritable and extensive serous tissue, spreading over its whole surface with great rapidity, is alone sufficient to account for the severity of the disease, especially when it is remembered that it occurs at a moment when the female system is always preternaturally irritable.

"On ordinary occasions, the well known local and general symptoms of inflammation are manifested, so that no doubt remains, as well of the nature of the affection, as of the proper remedies, but when endemic in hospitals, its apparent character is changed—diagnosis is difficult, and the anxious physician, alive to all the responsibilities of his situation, finds the most efficient antiphlogistic remedies unavailing, and even positively injurious in a disease essentially inflammatory. The following circumstances may be mentioned as more or less peculiar in the cases under consideration.

"1. The disease was not ushered in by any decided chill, often no cold sensations were experienced; and the subsequent phenomena render it probable that inflammation generally commenced on delivery, although no symptoms were detected before the second or third day.

"2. The almost entire absence of fever, that is, of a hot, dry skin, dry tongue, and other indications of the stage of excitement; the surface being usually pallid, with a livid tinge of the lips, cheeks, tongue, nails—also moist, and natural as to temperature. There were some exceptions to this remark, but the observation is correct as regards the most acute cases of the complaint—Case V. Pitney's; Case VI. Munhollan's.

"3. The pulse was never corded nor tense. The last patient, Case VIII. Sullivan, had slight tension of the pulse, but not to such a degree as on ordinary occasions to render the lancet essential. In all cases the pulse was at first rather large, soft, and compressible, not calling for, and even forbidding depletion; it was invariably frequent, at once rising to 120, and often could not be numbered towards the termination of the disease.

"4. The respiration was much hurried; in one patient, Case II. this was remarkably the case; like that of a person agitated, or exhausted by severe exercise. Connected with this, there were generally, almost universally, a disposition to blueness of the lips, and other evidences of imperfect hematosis.

"5. Perhaps, however, the greatest peculiarity of this epidemic was the slight degree of pain experienced. Riley, Case I. suffered the most; yet on the first day she made no complaint of pain; on the second, suffered only when bowels were moved; on the third, had severe pain, but it was completely relieved by V. S. and leeches; on the fourth, it returned and extended to the joints of the extremities, and was greatly aggravated by pressure. In no other patient was pain a prominent symptom; in some it was only produced by pressure, or by motion; and in two patients, Lehy and Borstall, no pain whatever was experienced, nor any tenderness on pressure during the whole course of an acute peritonitis!!

"The tympanitic condition of the intestines was much less than usual, particularly in those cases in which there was little or no pain.

"6. The almost universal existence of a furred and yellow, ('bilious,') tongue, indicative of visceral disorder, should perhaps be mentioned as a peculiarity. It was found in all the severe cases, and in nearly all who were delivered during the prevalence of the complaint. This derangement of the digestive functions, conjoined with a very irritable condition of the tissues, seemed to constitute that state of the system or organs which predisposes to peritonitis puerperalis, and which, under other exciting causes, as wounds, fractures, &c. gives rise to erysipelas. If so, the frequent conjoint prevalence of puerperal fever and erysipelas is, in some degree, explained—their predisposing causes being the same.

"7. The secretion of bile after the first few days became excessive, and appeared to be the exciting cause of the diarrhœa and vomiting, which were injurious and exhausting. Whether this increased secretion of bile depended on the disease, or on the measures adopted, particularly the exhibition of mercury, or on both, is a question not easily answered.

"These peculiarities will explain the difficulty of diagnosis—the inefficiency of the established modes of treatment, and the consequent fatality of the disease.

"1. *Difficulty of diagnosis.*—It may be safely asserted, that without the assistance of morbid anatomy, the nature of this disease would have been altogether inscrutable. What practitioner, however experienced in clinical observations, but ignorant of what the scalpel has revealed post mortem of the consequences of this affection, would have suspected a most rapid, acute, and extensive inflammation, involving the whole surface of the irritable and sensible serous membrane of the abdomen, when his patient complained merely of weakness; had no pain, nor even tenderness on pressing and handling the abdomen; no tympanitis; no purging; no dryness nor heat of surface; no tension nor quickness of pulse? The fact that authors have alluded to such cases, would afford but little assistance to the practitioner. In the case of Lehy, for example, the symptoms of disease were imperfect hematosis; rapid respiration, and great frequency of pulse, with diminution of the lochiæ; want of mammary secretion and great sense of debility; symptoms sufficiently decided to indicate dangerous internal disease, but whether of serous or mucous tissues in the abdomen or the thorax, are points which could not be positively determined. The last-mentioned symptoms however, judging from what occurred at the hospital, should, under similar circumstances, be considered as proof of the existence of acute peritonitis, for death unfortunately afforded the opportunity of ocular demonstration.

"2. The inefficiency of the established modes of treatment was too certainly exhibited at this period; confirming not only the experience of my predecessors in the hospital, but that also of accoucheurs to lying-in establishments generally. It may be useful to state what I believe to be the result of our experience in the different remedies employed, and then to deduce the practice which would promise most under similar modifications of peritonitis.

"1. *Blood-letting.*—General bleeding was tolerated in some of the cases, particularly in the last one, Sullivan's, where there was some tension of the pulse. It usually moderated, for a few hours, the local symptoms; but as a general observation, it was of no permanent benefit to the local affection, and was often decidedly injurious, by exhausting more rapidly the vital powers of the patient, as indicated by the increased frequency and weakness of the pulse, pallor of the surface, and sensation of weakness.

"The same remark was often applicable to leeches, as always moderating, and in one instance, Viney's, apparently subduing the local symptoms, but diminishing the general strength, so that the local affection appeared to spread with much more rapidity. In one case, Munhollan's, no relief was afforded, and the patient complained of being more uncomfortable. On the whole, leeches proved useful, but the evacuation of blood in whatever way effected was injurious when the pulse was rendered softer and more frequent. 'As salutary as these evacuations may prove in such cases, (of sporadic peritonitis,) exactly in the same ratio do I consider them injurious in epidemic peritonitis, especially in hospitals—peritonitis from internal causes. In this latter case, so far from procuring relief, they are ordinarily followed by great frequency and smallness in the pulse, a more evident alteration of the features, and increased swelling of the abdomen.'* Exceptions occurred to this rule of Baudelocque's, but none which would not confirm the observation that sanguineous evacuations are injurious when the pulse does not sanction their employment.

"2. *Emetics* were employed occasionally to relieve the stomach of any irritating ingesta, food, bile, &c. but were not relied upon as the efficient means in the management of the disease.

"3. *Purging*, as a plan of treatment, failed entirely: in all cases, whether excited spontaneously, or by medicine, it was very distressing and exhausting to the patient, with no apparent amelioration of the

* Baudelocque, Jr. on Puerperal Peritonitis, p. 346, Amer. Ed.

symptoms. At the commencement, however, laxatives or enemas to empty the bowels were grateful and beneficial.

"4. *Diaphoretics* were of no utility—indeed the patient often perspired with even a natural temperature of the skin, and yet experienced no relief.

"5. *Mercury.*—Much was anticipated from the use of this medicine, so valuable in inflammatory affections, especially of serous tissues. Hence, next to blood-letting, our main dependence was placed on its free exhibition; it was resorted to in every case, but on review, I cannot affirm that any good was effected, and perhaps it was even positively injurious. Salivation could not be induced: even in the comparatively protracted case of Borstall, where mercury was early, freely, and perseveringly employed, no genuine ptyalism was excited. An aphthous ulceration of the tongue, lips, &c. occurred without swelling of the face, ulceration of the gums, or any inflammation of the salivary glands, and without any mercurial fœtor. The patient appeared to sink more rapidly from this extensive irritation, and from the severely painful affection of the muscles of the forearm, which may possibly have been dependent on the mercurial influence. In Munhollan's case, which was fatal, and in Sullivan's, which was one of recovery, the great distress was from purging of bilious and serous fluids, apparently from the use of calomel; indeed, in all cases, it was disposed to act on the secretions of the abdominal viscera, rather than on the mouth, and had no influence in moderating the disease. Was the increased bilious evacuations in the latter stages of the complaint owing to the inflammation, or the mercury?

"6. The *oil of turpentine* was tried, but always excited vomiting, and was therefore abandoned.

"7. *Revulsives.*—Fomentations and poultices to the abdomen, extremities, vulva, and mammæ; hot pediluvium; oil of turpentine to body and limbs; warm and mucilaginous injections into vagina and rectum; blisters to the extremities and sometimes to the abdomen, were severally employed, and generally with advantage as secondary remedies. Moist heat, however applied, was generally grateful; but of course had, in so severe a disease, no very decided influence. The blisters were often detrimental, by increasing the restlessness of the patient, and preventing sleep. Nothing seemed to be gained by attentions to the mammæ; the application of plasters and poultices or of the child, did not seem to retard the secession of the milk; as the patient was disturbed by the infant, it was soon withdrawn.

"8. *Tonics and stimuli* were usefully employed, but not with any idea of curing the disease as has been mentioned, but on another principle presently to be indicated.

"9. *Cold.*—The great disappointment experienced in not being able to resort freely in this acute form of peritonitis to general and local bleeding, and in the efficiency of other remedies, early induced me to think of ice to the abdomen as the most promising means for arresting this terrible disease. To this practice I was led by principle, and by experience in analogous affections. For several years, I have taught at the Philadelphia Medical Institute, that the direct influence of cold is universally sedative, as respects the organic actions, to internal as well as to external capillaries, maintaining that cold applied to the skin does not determine the blood directly to the internal capillaries, but on the contrary, that the same condition of the capillary tissue is induced internally as well as externally, differing merely in degree. It may be useful to add, that the prominent facts on which this opinion of the modus operandi of cold is founded, are its acknowledged and powerful influence in moderating and arresting internal as well as external hæmorrhages; and also the decided benefit so universally experienced by its employment in many deep-seated inflammations of the limbs, of the brain, &c. when of the most acute character. Why then should it not be equally useful in acute peritonitis? I could not detect any satisfactory objection, but on theoretical views, and from experience in all analogous affections, I felt strongly inclined to use it freely and boldly. To this I was still further encouraged, not only by the failure of established modes of treatment, but by the accounts given of two cases of recovery in the Journal of the Royal Society of Medicine of Toulouse in 1827,* where ice was applied, and by the reports of several practitioners in favor of cold cataplasms, cold affusions, &c. In consultation, therefore, with my colleague, Dr. Lukens, it was agreed to employ ice in the next case that appeared suitable. That no bad consequences should result, if possible, from its employment, it was not resorted to until the symptoms were decided, until it was evident that sanguine evacuations could not be further employed, and until the lochiæ and the mammary secretion had ceased. These circumstances existing in Case VI., Munhollan's, ice in cloths and in bladders was applied to those portions of the abdomen which were tender on pressure, while dry heat, blankets, &c. were directed to the extremities, and calomel was freely exhibited with the views already explained. The result was not positively for or against its employment, but to me it was decidedly encouraging; for although the patient died as speedily as in the other cases, yet she was naturally of a delicate constitution, had been the subject of dysentery and bronchitis before and during her confinement, was delivered prematurely of twins, and was at the time the ice was applied much exhausted, (pulse being 144 and very weak,) by the disease and by the leeches, which last appeared to be injurious to the local as well as general symptoms. She had also hypertrophy of the left ventricle of the heart. The ice was agreeable to her feelings, far more so than the turpentine and other revulsives previously employed; it contributed greatly to the relief of her restlessness; moderated the local symptoms, and apparently arrested the progress of the inflammation; for, if any correct judgment could be formed from the symptoms, particularly the tenderness on pressure, before and after its application, of the extent of the inflammation, it did not extend on the peritoneum

* Vide North American Medical and Surgical Journal, vol. vi. p. 198. Archives Generales, vol. xvi. p. 136. Baudelocque on Puerperal Peritonitis, p. 400, Am. Ed.

after resort to the ice. *Dissection exhibited a more circumscribed inflammation than in either of the other fatal cases;* the effusion of lymph, &c. being confined to the lower portion of the abdomen, but in the other cases was found over all the viscera, liver and stomach not excepted.

"Was not this patient also injured by the calomel, which kept up a distressing purging, and excited no affection of the mouth and salivary glands?

"These observations certainly do not afford much encouragement in the treatment of peritonitis puerperalis as modified by epidemic causes, but by exhibiting the difficulties of the case may lead to a more scientific and efficient mode of treatment.

"Puerperal fever in the Pennsylvania Hospital was manifestly an acute inflammation of the peritoneum, conjoined with derangement of the chylopoietic viscera, and occurring in individuals whose systems were preternaturally irritable, and whose vital powers immediately yielded to the severity and extent of the local inflammation.

"The important and essential indication is to subdue the local disease, but its fulfilment was rendered fearfully difficult by the prostrated condition of the system. Hence, as frequently intimated, sanguineous depletion, purging, and other anti-phlogistic remedies were often detrimental, although temporary relief might be afforded. The general principle I would therefore deduce, is that the vital powers should not be enfeebled under the expectation of curing the local inflammation. If, therefore, the pulse becomes more irritable and frequent from the loss of blood, such evacuations should be abandoned, however apparently useful in moderating the local symptoms, and measures for supporting and invigorating the powers of life, be adopted. This I believe to be a principle of very universal application, but too often, especially at the present day, forgotten—for practitioners, from a laudable desire to overcome the local inflammation, the origo malorum, not unfrequently sacrifice the general strength of the patient.

"Anti-phlogistic remedies have seldom any other effect than simply to diminish, by a direct or indirect influence, the morbid excitement; the alteration of action, the change from a diseased to a healthy state, is effected almost exclusively by the natural powers of the economy, and demands therefore that a certain degree of power should be preserved—patients may be rendered so weak that very slight local disease may destroy them. Surgeons are familiar with these principles, and daily act upon them in the management of suppurating wounds, compound fractures, &c. of long standing, often employing stimulating measures, locally as well as generally.

"In all doubtful cases, the first evacuation of blood, if the pulse do not forbid the trial, should be tentative, as advised by Baudelocque, Jr. If the pulse acquire increased volume and tone, and become slower, the evacuations have been useful, and may sometimes be advantageously and even freely repeated. All direct depletion, however, must be effected within the first few hours; evacuations afterwards can seldom be tolerated, but moderate nourishment, tonics, and even stimuli, are demanded, that the patient may not sink from the local affection. These invigorating measures do not apparently augment the local affection.

"Sanguineous evacuation being forbidden, or having been carried as far as practicable, the patient must be trusted to local anti-phlogistic remedies, operating as sedatives or revulsives, with appropriate attentions to the general system. Judging from the few cases above detailed, and for reasons already advanced, I would prefer the sedative influence of ice to the abdomen as more promising than any other measure hitherto recommended. It certainly deserves a more enlarged and decided trial, having been apparently successful in some few cases on record—being more grateful to the sensations of the patient, and certainly exciting a more powerful influence, whether for good or evil, than any other measure at command. In using it, moderate warmth should be preserved in every other portion of the surface, the bowels be kept at rest by anodynes, the general strength supported by simple unirritating food, tonics, or even stimuli; and any occasional or accidental symptom be appropriately attended to. Should circumstances forbid the use of ice, revulsives alone remain at our command, and should then be resorted to decidedly: a blister to cover the whole anterior and lateral portions of the abdomen can alone be adequate to the emergencies of the case; small blisters are inadequate as revulsives, and probably produce as much general distress as the large one over the abdomen. Although my confidence in mercury is destroyed as a remedy for peritonitis, it perhaps should not be entirely abandoned, as in small and repeated doses with opium, so as to prevent any cathartic effect, it may correct the derangement of the chylopoietic viscera, the integrity of whose functions is so exceedingly important in maintaining the vital powers of the system.

"Bleeding, general and local, regulated entirely by the condition of the pulse, especially as regards tension, with the constant application of ice to the abdomen, appear, therefore, to be the most promising remedies for puerperal peritonitis, under the direction of a well-instructed and attentive practitioner."

We have never yet seen puerperal fever epidemic, nor of a typhoid type. In Cincinnati, it has constantly, as far as we have seen, presented a simple inflammatory character, and required a pure anti-phlogistic treatment. Blood-letting, antimonials conjoined with calomel, calomel alone in large doses, calomel and opium, warm fomentations to the abdomen, and a large blister, have been our remedies, and invariable success has followed their application. The disease has generally been ushered in with a chill, but a case now under treatment made its onset without the slightest rigor. Hysterical symptoms are often present, apparently from the involvement of the uterine system in the inflammation. The physician should not suffer them to mislead him in the treatment.

